# Physics-Informed Simulated-Annealing Path-Planning for Mitigating Thermal Accumulation During Nanosecond Laser Drilling of PMMA Micro-Hole Arrays

**DOI:** 10.3390/mi17101172

**Published:** 2026-10-08

**Authors:** Jing Liu, Shangkai Chen, Mian Zheng, Yimin Feng, Liwang Zheng, Guanglu Wei, Ming Li

**Affiliations:** 1 School of Computer Science (School of Artificial Intelligence), South-Central Minzu University, Wuhan 430074, China; 2 Bayannur Power Supply Company, Inner Mongolia Power (Group) Co., Ltd., Bayannur 015000, China; 3 China Ship Development and Design Center, Wuhan 430064, China

**Keywords:** laser processing, simulated annealing algorithm, path optimization, thermal accumulation, PMMA micro-drilling

## Abstract

The high-precision laser micromachining of thermally sensitive PMMA polymers is severely limited by thermal accumulation, which deteriorates the surface quality of micro-hole arrays. Current laser drilling path optimization strategies predominantly prioritize geometric efficiency by minimizing travel distance, whereas quantitative thermal-constrained path optimization for PMMA micro-hole drilling remains underexplored. This work proposes a constrained simulated-annealing (SAA) path-planning strategy that embeds physical thermal constraints (critical thermal radius R and spacing threshold M) into the classic meta-heuristic algorithm, effectively suppressing transient thermal superposition between adjacent micro-holes while maintaining acceptable machining efficiency. Compared with the conventional sequential scanning mode (SSM), the SAA strategy reduces the molten deposit width around micro-holes by 22.3% under identical machining conditions (3 W laser power, 60 mm/s scanning speed, and a single scanning pass). A inherent trade-off is also observed: the thermally isolated jump path increases the total travel distance, resulting in approximately 1.3-fold longer machining time. Benefiting from the suppressed thermal accumulation, the SAA path effectively eliminates thermal-induced micro-hole coalescence at a compact hole spacing of 50 μm, yielding well-defined micro-hole contours and enabling the reliable fabrication of high-quality E-shaped structural color patterns with tunable optical responses under natural light.

## 1. Introduction

With the rapid development of the manufacturing industry, there has emerged a heightened demand for accuracy and quality in micro-scale structures [1]. Polymethyl methacrylate (PMMA) [2], characterized by its stable chemical properties, low damage threshold, excellent optical properties and low cost, has widespread applications in micromachining industries, such as microfluidic chips [3,4] and microchannel emulsification plates [5]. The performance of these structures is intimately tied to their processing quality [6,7]. However, conventional mechanical machining can introduce some defects, like burrs, stratification and severe tool wear, which seriously influence product quality and elevates subsequent processing costs [8,9]. Therefore, some non-conventional processing methods, such as electrical discharge machining [10,11], abrasive water jet machining [8,12,13], the chemical etching method [14,15], the focused ion beam method [16,17] and laser machining [18,19,20], have gradually attracted significant attention. Among these, laser processing [21,22] stands out due as it is non-contact and high-efficiency, has high precision and high cost-effectiveness, does not lead to tool wear and is not limited to particular material types. It has become a popular micromachining method. Nevertheless, the inherent thermal accumulation effect of laser processing inevitably induces microcracks, poor surface roughness and residual thermal defects, which severely degrade the structural integrity of thermally sensitive PMMA materials [23].

Several scholars have explored the impact of various laser processing parameters, including hatching distance [24], pulse repetition rate [25], laser source types [26,27], pulse duration [28] and processing environment [29,30]. They demonstrated that appropriate processing parameters can positively influence the quality of laser processing. Notably, the laser processing path also plays a crucial role in determining processing quality. Extensive research has been conducted on path optimization in this regard. Kim et al. [31] used a heuristic back-tracking method to find the near-optimum cutting path considering the minimum heat effect on deformation. They utilized a micro-genetic algorithm to determine the minimum path distance before evaluating the temperature of the next piercing point. Li et al. [32] proposed an interlaced scanning mode (ISM) to improve surface quality based on the default sequential scanning mode (SSM) of the top-down multi-layer concentric circle-filling scanning process. Compared to SSM, the overall average width of the heat-affected zone (HAZ) of the ISM is reduced by 25.85%, and the damage degree of the HAZ and its vicinity in ISM is less than that in SSM. Ye et al. [33] used a picosecond laser to drill holes under three different scanning paths (rotary cutting, parallel filling cutting and cross-filling cutting). The experimental results revealed that the laser rotary cutting method had the highest material removal efficiency and the smallest taper angle. Makbul Hajad et al. [34] proposed an approach aimed at minimizing both the cutting path and heat accumulation during the laser cutting process. Their algorithm was based on a memetic algorithm combining a powerful genetic algorithm with an adaptive large neighborhood search. In their study, the cutting path problem was modeled and solved in accordance with the generalized traveling salesman problem. Zhang et al. [35] developed a processing-time evaluation model, and introduced an adaptive discrete grey wolf optimization (A-D-GWO) algorithm to optimize the shortest path. The experimental results demonstrated that the A-D-GWO algorithm had better stability and accuracy compared to conventional algorithms. Existing path optimization methods fall into heuristic geometric scanning and meta-heuristic intelligent algorithms. Fixed interlaced scanning lacks a quantitative thermal constraint judgment; genetic, grey wolf and memetic algorithms prioritize the shortest distance while weakening thermal suppression, with few studies quantifying heat-affected zone as a hard path constraint. Notably, Yulong Ding et al. [36] established a physical-calibrated machine learning framework for laser processing optimization, verifying that integrating practical physical constraints can effectively compensate for the deficiencies in traditional purely data-driven and geometry-oriented optimization methods, and significantly improve processing controllability and structural consistency. Most research focuses on metal/sapphire, while path planning for thermally sensitive PMMA polymer remains insufficient.

To fill this gap, we customize a constrained simulated-annealing algorithm (SAA) for PMMA hole array laser drilling. Classic SAA is adopted as the optimizer, and thermal-related hard constraints (critical thermal radius *R* and spacing point number *M*) are integrated into path iteration to reduce thermal superposition. A series of laser drilling experiments are carried out to compare hole quality under SSM and constrained SAA paths. The parameter window where path optimization works effectively is identified, and the trade-off between surface quality and machining efficiency is quantitatively analyzed.

## 2. Path-Planning of Thermally Constrained Simulated-Annealing Algorithm

### 2.1. Thermally Constrained Simulated-Annealing Path Planning

Our processing objective is to obtain an array composed of *n* × *m* holes on PMMA (Shenzhen Bangbaite Industrial Co., Ltd., Shenzhen, China); each hole is etched by a laser following a circular trajectory path. After completing one hole, the laser head moves a specific travel distance to begin etching the subsequent hole. This sequence is repeated to form the desired hole array. All algorithms were implemented in MATLAB (Version R2022b, MathWorks, Natick, MA, USA).

The laser processing path refers to the trajectory traveled by the laser head from the starting point, through the processing of all components, and back to the starting point. It can be expressed as *Path* = (*Ps*_1_, *L*_1_, *Ps*_2_, *L*_2_ … *P_mn_*_−1_, *L_mn_*_−1_, *Ps_mn_*), where *Ps*_1_ is the etching starting point of the first micro-hole, *L*_1_ is the length of the flight path from the first micro-hole to the second micro-hole, *Ps_mn_* is the etching starting point of the last micro-hole, and *mn* denotes the total number of required micro-holes. The flying distance of the laser head refers to the sum of the path lengths for the laser head moving between each hole after switching off the laser beam during the machining path. The total flying distance can be expressed as *L* = *L*_1_ + *L*_2_ + ⋯ + *L_mn_*_−1_. Shortening the flying-path length reduces machining time and energy consumption. After etching a hole, residual heat diffuses outward; if the next hole is etched nearby, it is significantly affected. Thus, thermal effects must be considered when sequencing hole positions.

SAA [37] is inspired by the principle of physical annealing, which can break through the limitations of the hill-climbing algorithm and escape from the local optimal solutions. The outer loop of SAA is a decreasing iteration of the dimensionless algorithmic temperature *E_k_* (*k* = 1, 2, 3, …), updated by *E_k_* = *E*_0_*B^k^*, where *E*_0_ is the dimensionless initial temperature, *B* is the dimensionless annealing constant, and *k* is the iteration time. The loop exits when *E_k_ < F*, where *F* is the dimensionless final temperature. In this study, *E*_0_ is set to 100, B is set to 0.95, and *F* is 70.

In the inner loop, the drilling path is updated at each temperature *E_k_* according to the Metropolis criterion [38]:

(1) $$P_{A_{k}}=\left\{\begin{array}{l} \;1\;\;\;\;\;\;\;\;\;\;\;\;\;\;\;\;\;\;\;\;\;\;\;\;\;\;\;\;\Delta L=L_{\mathrm{new}}-L\leq 0\\ \;\exp(-\frac{\Delta L/L}{E_{k}})\;\;\;\;\;\;\mathrm{others} \end{array}\right.$$

where Δ*L* is the difference in the overall travel distance between the new generated path *L*_new_ and the current path *L*. When Δ*L* ≤ 0, the drilling path is improved and the new path is accepted with the probability of 1. When Δ*L* > 0, the new path is accepted with a limited probability, which can escape from the local optimal solutions and avoid fast convergence.

In contrast to conventional simulated-annealing solvers, which merely minimize traveling distance, the physics-informed simulated-annealing algorithm adopted herein incorporates two types of thermally motivated hard constraints: the analytically estimated dimensionless critical thermal radius *R* and the physically inspired sequential–discrete spacing-point number *M*. Rather than setting thermal accumulation as an independent optimization objective, these thermal-physics-derived constraints are enforced throughout path iterations to suppress thermal superposition in PMMA laser drilling. The spatial constraint *R* forbids immediate drilling within the critical thermal zone surrounding a previously machined hole. Meanwhile, the sequential constraint *M* demands at least *M* intermediate drilling points before revisiting such thermally risky regions, extending the effective cooling interval for subsequent re-processing.

These two constraints prevent thermal residual from previous holes from degrading subsequent hole quality. With total flight distance *L* as the efficiency metric, the path optimization problem is formulated as

(2) $$\left\{\begin{array}{c} \mathrm{minL}=\min\sum_{i=1}^{\mathrm{mn}-1}\sqrt{{\left(x_{\left(i+1\right)}-x_{i}\right)}^{2}+{\left(y_{\left(i+1\right)}-y_{i}\right)}^{2}}\\ \sqrt{{\left(x_{\left(i+b\right)}-x_{i}\right)}^{2}+{\left(y_{\left(i+b\right)}-y_{i}\right)}^{2}}>R,\;\;\;\;\;\;\;b=1,2,\ldots M \end{array}\right.$$

For nanosecond laser drilling of PMMA, laser energy is deposited within nanosecond pulses; subsequent heat conduction dominates thermal diffusion after pulse termination. The thermal diffusion characteristic length *L_th_* is defined as

(3) $$L_{\mathrm{th}}=\sqrt{4\alpha t}$$

where *α* is the thermal diffusivity of PMMA (*α* = 1.12 × 10^−7^m^2^/s) and *t* = 1.25 ms denotes the cooling interval between the successive adjacent drilling actions of the galvanometer scanner. Accordingly, *L_th_* is calculated to be approximately 748 μm. Normalized by the hole spacing *s*, the dimensionless critical thermal radius yields *R* = *L_th_*/*s* ≈ 3. This spatial constraint prohibits immediate drilling inside the thermally risky zone surrounding the previously machined hole to avoid direct spatial thermal superposition.

Unlike *R*, *M* cannot be analytically derived from continuous heat-conduction equations, since the discrete sequence counter does not strictly map to physical cooling time under variable galvanometer jump durations. The trend plot of minimum flying distance *L* versus *M* is provided in Supplementary Materials S1. Balancing thermal-suppression capability and additional flying-distance overhead, *M* = 2 is selected as the optimal setting for the present configuration. Under these combined constraints, the algorithm produces an optimized drilling sequence with low thermal accumulation and moderate travel overhead within 60 s of computation.

### 2.2. Experimental Materials, Equipment and Setup

The material selected in this experiment is a black PMMA square plate with a thickness of 0.3 mm and a side length of 40 mm. This bulk-modified PMMA is uniformly incorporated with carbon black particles during polymerization and molding, rather than being dyed or spray-coated. Unlike pure transparent PMMA, which barely absorbs 1064 nm laser light, the embedded carbon black fillers efficiently capture incident laser photons and convert light energy into heat, which greatly enhances the near-infrared absorption capability at 1.06 μm and enables effective laser micro-processing of PMMA.

**Figure 1:**
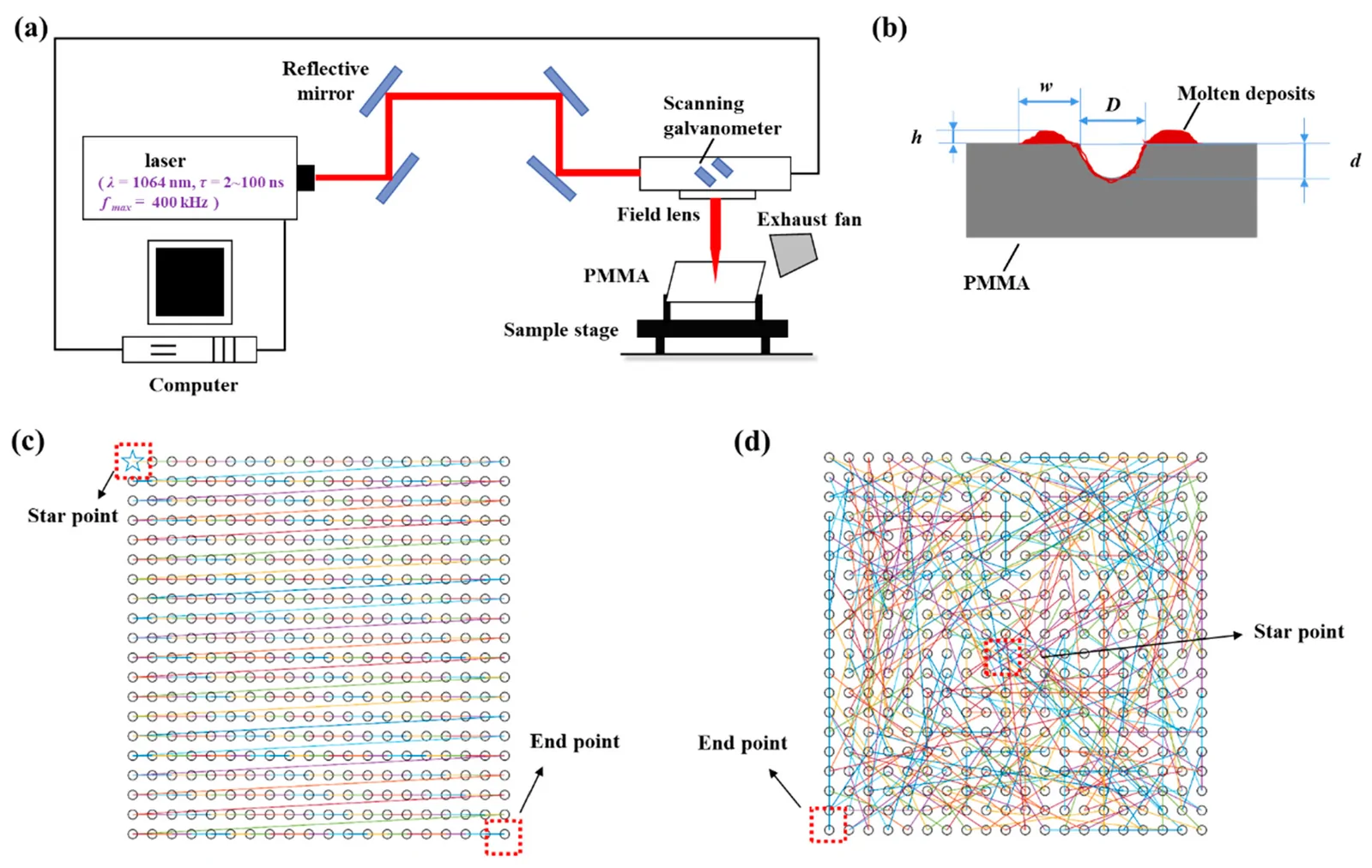
Schematic diagrams of (**a**) the laser processing system and (**b**) the hole quality parameters (the height *h* and width *w* of the molten deposits, and the depth *d* and diameter *D* of a single hole). (**c**) SSM path and (**d**) SAA path (*R* = 3, *M* = 2) of the 20 × 20 hole array. The five-pointed star denotes the start point, and the dashed red box marks the end point.

The experiments are conducted on a nanosecond laser processing system equipped with a scanning galvanometer (three axes hybrid laser engraving machine MD-X1000 (KEYENCE Company, Osaka, Japan), with a wavelength *λ* = 1064 nm, pulse frequency *f* = 0~400 kHz, maximum output power *P_max_* = 30 W and pulse duration *τ* = 2~100 ns). The schematic diagram of the experimental setup is shown in Figure 1a. Before the experiments, the sample surface is wiped by dust-free mirror-wiping paper moistened with absolute alcohol. This step ensures the removal of dirt and particulate impurities. Subsequently, the material is positioned on the processing platform and etched by the laser processing system under the drilling paths of a Computer-Aided Design (CAD) drawing. During the experiments, the exhaust fan should be opened to pump away the smoke, and all finished samples must undergo ultrasonic cleaning for approximately 120 s to effectively eliminate debris resulting from the etching process. Four parameters characterizing hole quality are introduced, including the height *h* and width *w* of the molten deposits around a single hole, and the depth *d* and diameter *D* of a single hole, as illustrated in Figure 1b. In this work, a 20 × 20 hole array was fabricated on PMMA with an adjacent hole spacing of *s* = 250 μm. The CAD-defined baseline of SSM and thermally constrained SAA path are presented in Figure 1c and Figure 1d, respectively.

## 3. Experimental Results and Analysis

Parameters including hole depth, hole diameter, and molten-deposit height are largely governed by single-point laser energy input and exhibit weak sensitivity to processing sequence. Relevant experimental results are provided in Supplementary Materials S2. The width of molten deposits is highly sensitive to inter-hole thermal superposition, rendering it a primary indicator for evaluating path-optimization performance. Accordingly, we focus on investigating the influences of laser power *P*, scanning speed *v*, and scanning number *N* on molten-deposit width *w*.

### 3.1. The Effect of P, v and N on the Width w of the Hole Array

**Figure 2:**
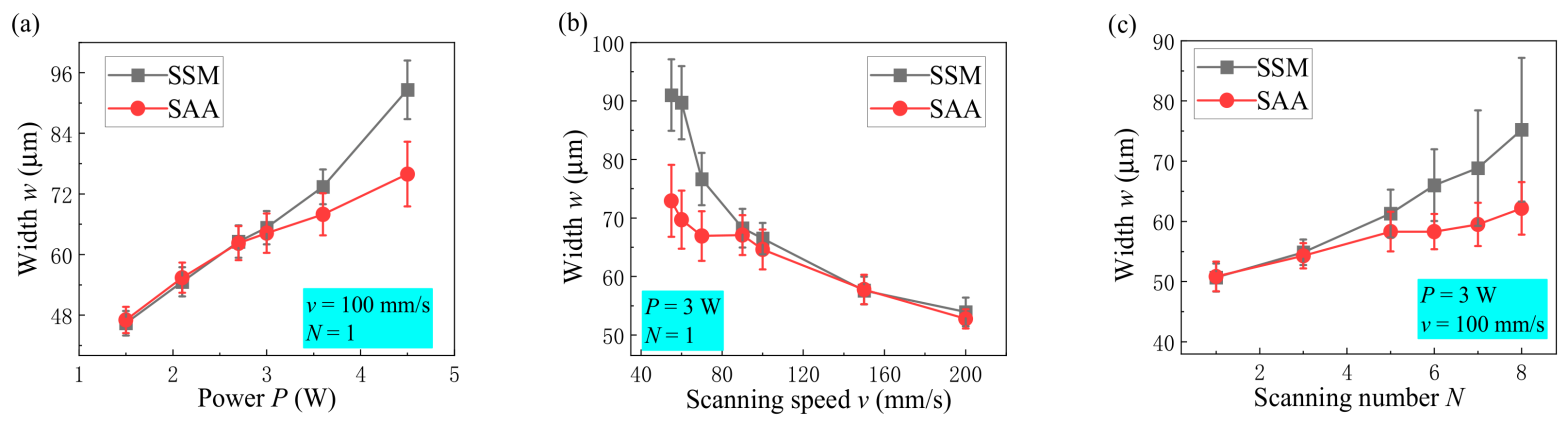
The molten deposits width *w* around holes as a function of (**a**) the power *P* at *v* = 100 mm/s and *N* = 1; (**b**) the scanning speed v at *P* = 3 W and *N* = 1; (**c**) scanning number *N* at *P* = 3 W and *v* = 100 mm/s.

In this section, Figure 2 presents the molten-deposit width *w* as a function of laser power *P*, scanning speed *v*, and scanning number *N* for the SSM and SAA scanning strategies. In Figure 2a, the molten-deposit width *w* shows negligible variation for *P* ≤ 3 W. For *P* > 3 W, the width *w* obtained with the SAA path is evidently lower than that of the SSM path. Table 1 displays the widths *w* of the molten deposits when laser power *P* = 3.6 W and 4.5 W. Notably, at *P* = 4.5 W, the width *w* under the SAA path can be reduced by about 18% compared to the SSM path. Although the single-hole laser power is identical for both approaches, their performance gap stems from thermal-effect accumulation. The SSM path suffers severe thermal superposition among adjacent holes. In comparison, successive holes in the SAA scheme are barely affected by thermal loads from previously drilled features. Consequently, SAA mitigates thermal buildup and generates narrower molten deposits.

As illustrated in Figure 2b, the molten-deposit width *w* decreases with increasing scanning speed *v* for both the SSM and SAA paths. This trend arises from the reduced laser-pulse overlap at higher scanning speeds [39], which decreases the effective energy density deposited on the material. Notably, the widths *w* remain comparable for *v* > 70 mm/s, whereas the SAA path produces distinctly narrower deposits under *v* ≤ 70 mm/s. Table 2 summarizes the corresponding width data for *v* ≤ 70 mm/s. At *v* = 60 mm/s, the SAA path reduces *w* by approximately 22.3% relative to the SSM path. For a given scanning speed, the incident laser energy is equivalent for both strategies; nevertheless, lower scanning speeds enlarge single-pulse energy overlap and aggravate thermal accumulation. Low scanning speeds inevitably lead to excessive heat accumulation inside the processing area, which is the fundamental reason for the significant difference in molten-deposit width between the two scanning paths at low-speed machining. The SAA path provides superior temporal and spatial conditions for heat dissipation, which suppresses molten-deposit expansion.

Figure 2c shows that molten-deposit width *w* gradually increases with scanning number *N* for both SSM and SAA. Such broadening is mainly caused by elevated areal laser energy density. For *N* ≤ 5, the widths *w* under both paths are similar. However, when *N* > 5, the width *w* under the SAA path becomes notably narrower than that under the SSM path. Table 3 presents the widths of the molten deposits when scanning number *N* > 5. Notably, when *N* = 8, the width *w* under the SAA path is reduced by approximately 17.4% compared to the SSM path. The reason for this is that the thermal energy accumulation is much greater when the scanning number continues to increase, leading to wider molten deposits in the processing area. However, when processing under the SAA path, the width *w* decreases due to the reduced accumulation of the thermal effect compared to the SSM path, eventually enhancing the processing outcomes.

### 3.2. The Surface Morphology Analysis of the Hole Array

**Figure 3:**
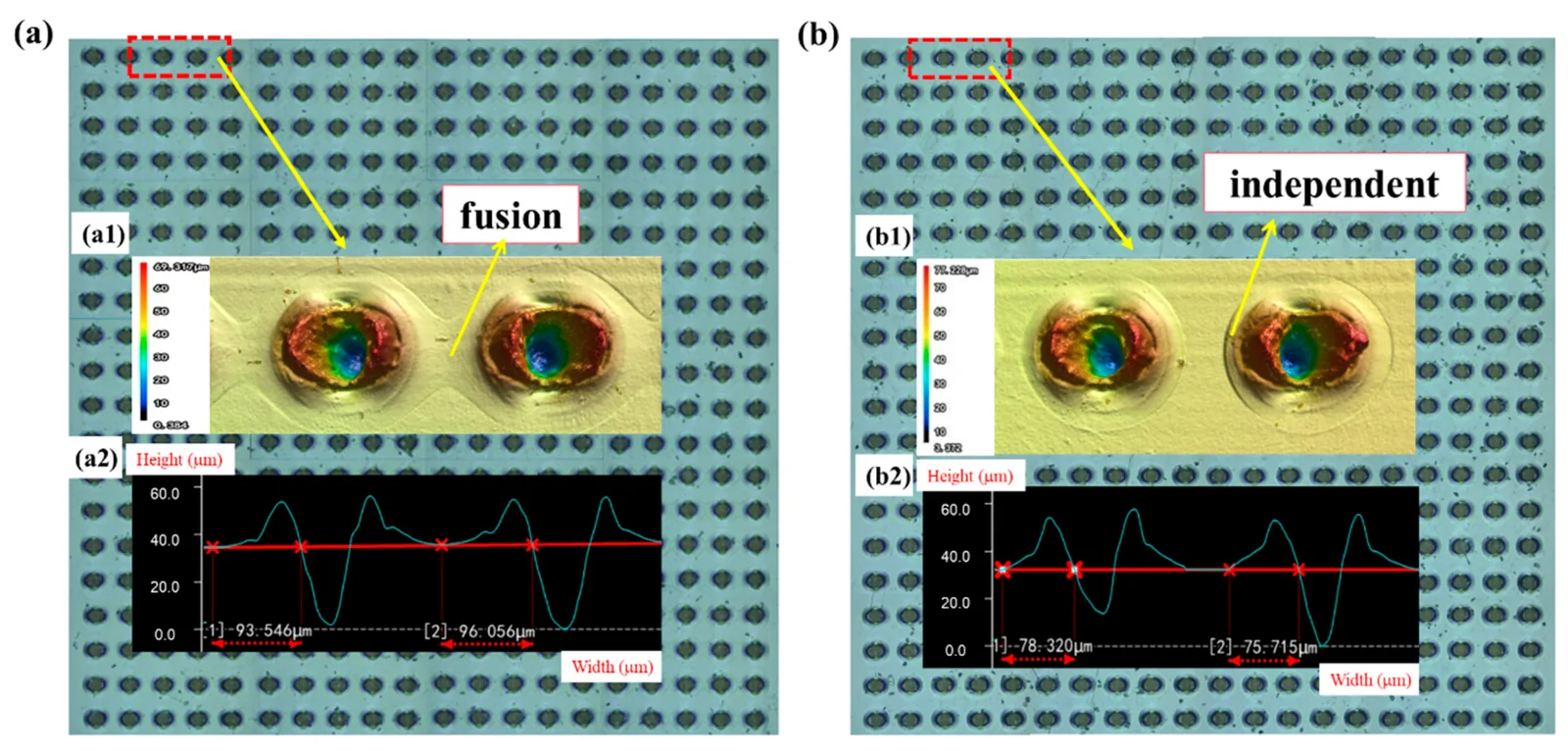
The surface morphology of the hole array at *P* = 4.5 W, *v* = 100 mm/s and *N* = 1 under the (**a**) SSM path, (**a1**) Enlarged 3D images, and (**a2**) cross-section profile of (**a1**) (horizontal axis represents width and vertical axis represents height). (**b**) SAA path, (**b1**) Enlarged 3D images, and (**b2**) cross-section profile of (**b1**).The green line is the height profile obtained via 3D confocal microscopy, the horizontal red line indicates the characterized measurement, and their numerical values are presented underneath.

During laser processing, incident laser energy is absorbed by the target material, inducing a sharp temperature rise within the irradiated zone and driving thermal conduction toward surrounding regions. In this section, the surface morphologies of the hole array fabricated via the two scanning strategies are characterized by a three-dimensional (3D) laser confocal microscope (VK-X250K of KEYENCE Company, Osaka, Japan). Figure 3a and Figure 3b respectively show the morphology of the hole array under the SSM and SAA paths at *P* = 4.5 W, *v* = 100 mm/s and *N* = 1. Holes machined by the SSM strategy exhibit obvious adjacent fusion, with an average molten-deposit width of approximately 94.80 μm. In contrast, the SAA path produces well-separated, intact hole structures with a reduced molten-deposit width of approximately 77.02 μm. For the SSM scanning sequence, the center-to-center distance between consecutive holes is approximately 250 μm. Residual heat from the preceding hole cannot fully dissipate before the next hole is machined, which degrades the quality of subsequent features. This cumulative thermal effect progressively exacerbates thermal buildup and ultimately causes adjacent-hole fusion. By contrast, the SAA path provides a minimum inter-hole spacing of 750 μm. Subsequent holes are located beyond the thermal-influence zone of previously drilled features. Accordingly, each hole is barely disturbed by thermal residuals from prior machining events, and thermal-effect accumulation is substantially suppressed relative to the SSM scheme. Consequently, the SAA path preserves discrete, intact hole geometries and yields improved machining quality.

Furthermore, the SAA strategy requires a longer machining time owing to its extended beam travel distance. Multiple timing measurements recorded by the DelphiLaser laser etching software version ASP-6483-Q (DelphiLaser Co., Ltd., Suzhou, China) reveal that the total processing duration for SAA is roughly 1.3 times that of the SSM scheme under the specific scanning speed and processing area adopted in this work. This ratio is not universally applicable and depends on the machining conditions. For small processing regions, overhead from extra turning motions dominates and may raise the time ratio. Similarly, adjusting the scanning speed changes the absolute machining time for both patterns, thereby shifting their relative time ratio.

### 3.3. Effect of Different Machining Paths on Structural Color

**Figure 4:**
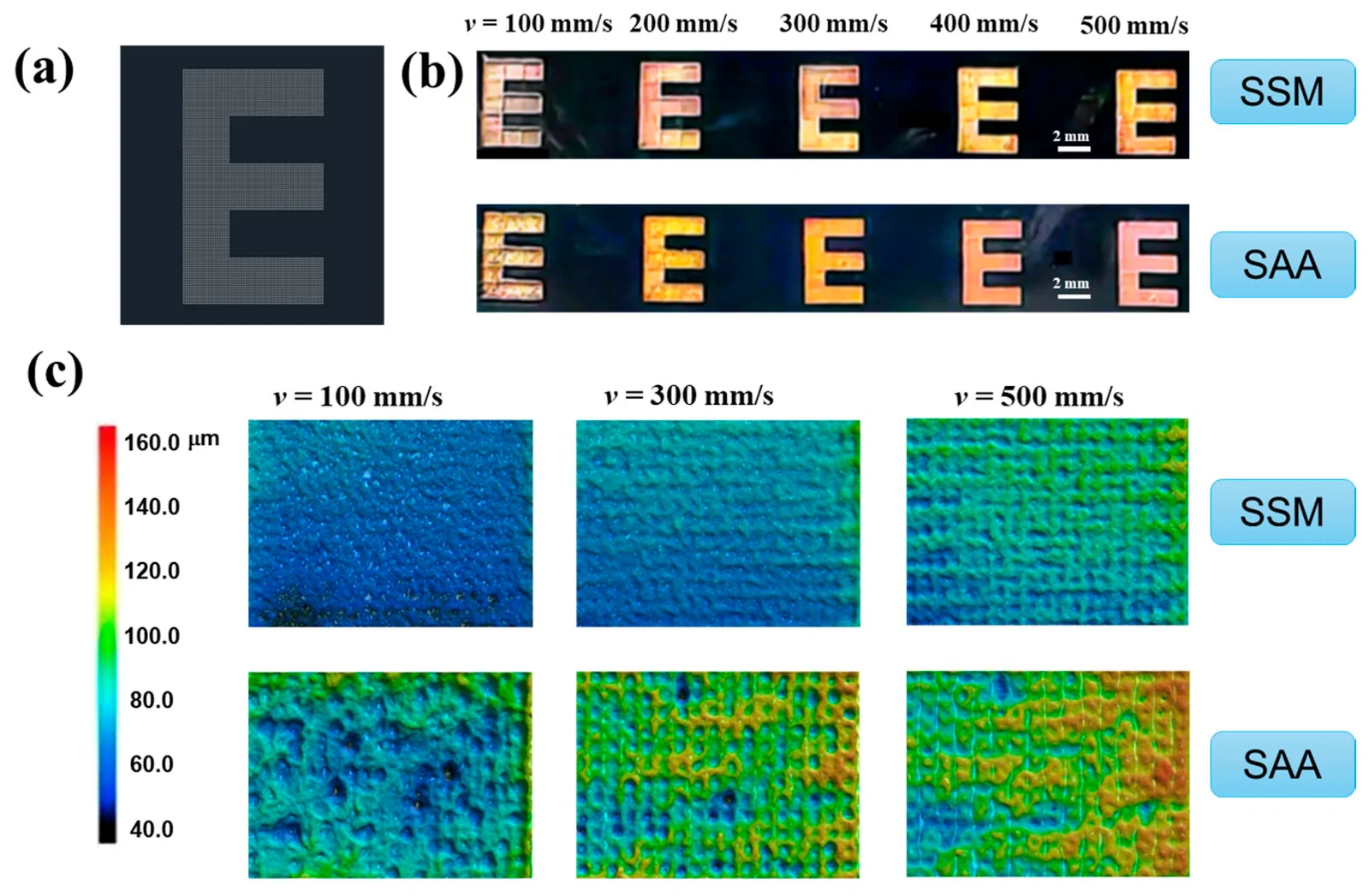
(**a**) Schematic of the letter-E pattern. (**b**) Optical photographs of the patterns fabricated by SSM and SAA strategies, captured under natural light. (**c**) 3D morphologies of the right-hand region of E-shaped structures machined via SSM and SAA strategies.

Based on the optimal constrained parameters derived in the preceding section, this subsection explores the optimized SAA scanning strategy’s structural-color generation capabilities. The pattern “E”, as illustrated in Figure 4a, was fabricated using both the SSM and SAA paths. For these experiments, the inter-hole spacing was fixed at 50 μm, together with *P* = 3 W and *N* = 1. The scanning speed *v* was varied at 100, 200, 300, 400 and 500 mm/s for pattern fabrication with the two paths. Optical photographs of the resulting E-shaped patterns captured under natural light are provided in Figure 4b. Distinct structural-color appearances are observed as a function of the scanning speed and scanning strategy. At *v* = 500 mm/s, the “E” structure machined by the SSM path exhibits a yellowish color, whereas the counterpart from the SAA path shows a reddish color. The corresponding three-dimensional topographies acquired from the right-side region of each E-pattern are presented in Figure 4c, revealing prominent morphological discrepancies between the two approaches. The surface discoloration of laser-etched PMMA originates primarily from chemical coloration induced by thermally degraded solid residues. Severe local heating further triggers secondary pyrolysis and oxidation, retaining solid residues on the etched surface, including oxidized polymer fragments with conjugated C=C bonds, carbonyl-rich oligomeric segments and oxygen-deficient amorphous nanocarbon particles. The conjugated π-electron systems of these residues absorb visible light and produce yellow-to-reddish coloration, with longer conjugated chains leading to deeper color tones.

The E-shaped pattern consists of densely packed micro-holes. The conventional SSM path induces severe thermal accumulation, and the small inter-hole spacing leads to the coalescence of adjacent micro-holes. As a result, individual micro-hole features are largely obliterated, forming a continuous, seemingly uniform molten surface. This pseudo-uniform morphology stems from excessive thermal melting rather than improved processing quality. In contrast, via SAA processing, the micro-hole contours remain well-resolved with distinct microscopic features. This indicates that the SAA-optimized path effectively mitigates thermal accumulation and reduces thermal superposition between adjacent processing points. Macroscopically, the SAA-processed surface presents a more uniform color in Figure 4b, further verifying its superior thermal regulation performance.

## 4. Conclusions

This paper develops a thermally constrained simulated-annealing (SAA) path-planning strategy by incorporating constraint conditions such as the critical radius *R* and spacing point number *M*, aiming to address the severe thermal accumulation issue of laser micro-drilling on thermally sensitive PMMA. The surface quality achieved under both the SSM and SAA paths is meticulously investigated and compared across various processing conditions. Compared to the SSM path, under processing conditions of *P* = 3 W, *v* = 60 mm/s and *N* = 1, the width *w* of the molten deposits under the SAA path can be reduced by approximately 22.3%. An inevitable efficiency trade-off is quantified: the thermally optimized discrete scanning path avoids thermal superposition but increases the total travel distance, resulting in a machining time approximately 1.3 times that of conventional SSM. Using the thermally constrained optimized SAA scanning strategy, E-shaped structural-color patterns were fabricated, preserving well-resolved hole contours and producing distinct structural-color responses under natural-light illumination.

By further refining and customizing the algorithm, the path optimization method has the potential to be implemented in the processing industry for applications that require minimized thermal-effect accumulation during processing. This also offers a valuable and meaningful reference for improving the manufacturing accuracy and quality of microscale structures. Nevertheless, as a modified scanning trajectory, the complex SAA path may introduce subtle dynamic variations for galvanometer scanning systems and potentially trigger fly-by instabilities, which may further affect the spatial marking precision under extreme or high-precision processing scenarios. Therefore, the dynamic scanning stability and spatial positioning accuracy of the SAA path will be systematically investigated in our future work, to further promote the practical industrial applicability of the proposed thermal-optimized scanning strategy.

## Figures and Tables

**Table 1:** The widths *w* at *P* = 3.6 W and *P* = 4.5 W.

	SSM (μm)	SSA (μm)	Reduction Rate
Width (*P* = 3.6 W)	73.43 ± 3.43	67.97 ± 4.16	7.4%
Width (*P* = 4.5 W)	92.64 ± 5.81	75.94 ± 6.39	18.0%

**Table 2:** The widths *w* at different scanning speeds *v.*

	SSM (μm)	SAA (μm)	Reduction Rate
Width (*v* = 55 mm/s)	91.01 ± 6.09	72.94 ± 6.14	19.9%
Width (*v* = 60 mm/s)	89.72 ± 6.26	69.71 ± 4.95	22.3%
Width (*v* = 70 mm/s)	76.64 ± 4.47	66.93 ± 4.23	12.7%

**Table 3:** The widths *w* at different scanning numbers *N.*

	SSM (μm)	SAA (μm)	Reduction Rate
Width (*N* = 6)	66.01 ± 5.96	58.30 ± 2.92	11.7%
Width (*N* = 7)	68.86 ± 9.57	59.50 ± 3.59	13.6%
Width (*N* = 8)	75.23 ± 11.91	62.17 ± 4.37	17.4%

## Data Availability

The raw data supporting the conclusions of this study will be made available by the corresponding author upon reasonable request.

## Supplementary Materials

The following supporting information can be downloaded at: https://www.mdpi.com/article/10.3390/mi17101172/s1, Figure S1: Schematic diagrams of SAA paths under different constraints *R* = 6. (a) *M* = 1; (b) *M* = 2.; Figure S2: Trend of minimum flying distance L against spacing-point number *M* (*R* = 6); Figure S3: At the conditions of the *v* = 100 mm/s and *N* = 1, the effect of laser power *P* on the hole quality parameters under the SSM and SAA paths: (a) The height h and (b) width w of the molten deposits around single hole; (c) The depth d and (d) diameter D of the single hole; Figure S4: At the conditions of the *P* = 3 W and *N* = 1, the effect of scanning speed *v* on the hole quality parameters under the SSM and SAA paths: (a) The height h and (b) width w of the molten deposits around single hole; (c) The depth d and (d) diameter *D* of the single hole; Figure S5: At the conditions of the *P* = 3 W and *v* = 100 mm/s, the effect of scanning number *N* on the hole quality parameters under the SSM and SAA paths: (a) The height *h* of and (b) width *w* of the molten deposits around single hole; (c) The depth d and (d) diameter *D* of the single hole.
