# Supplementary material for: Physics-Informed Simulated-Annealing Path-Planning for Mitigating Thermal Accumulation During Nanosecond Laser Drilling of PMMA Micro-Hole Arrays
Source: Micromachines (Basel). 2026 Oct 8;17(10):1172. doi: 10.3390/mi17101172 (PMC13707894; doi:10.3390/mi17101172)
Supplement: Supplementary file 1 [file micromachines-17-01172-s001.zip › micromachines-4575667-supplementary.pdf]

Supplementary Materials

Thermal-constrained Laser Drilling Path Planning for PMMA Hole Arrays Based on Customized Simulated Annealing Algorithm

Jing Liu[1*], Shangkai Chen[2], Mian Zheng[3], Yimin Feng[1], Liwang Zheng[1], Ming Li[1*]

[1] School of Computer Science (School of Artificial Intelligence), South-Central Minzu University, Wuhan 430074, China

[2] Bayannur Power Supply Company, Inner Mongolia Power (Group) Co. LTD, Inner Mongolia 015000, China

[3] China Ship Development and Design Center, Wuhan, 430064, China

* Corresponding authors： jingliu@scuec.edu.cn

S1. The relationship of the minimum flying distance with spacing-point number *M*

When $R = 6$ is fixed, varying M produces distinct SAA trajectories. Figure S1 compares the SAA paths obtained for $M = 1$ and $M = 2$. A larger M results in more complex trajectories and greater flying-path length.

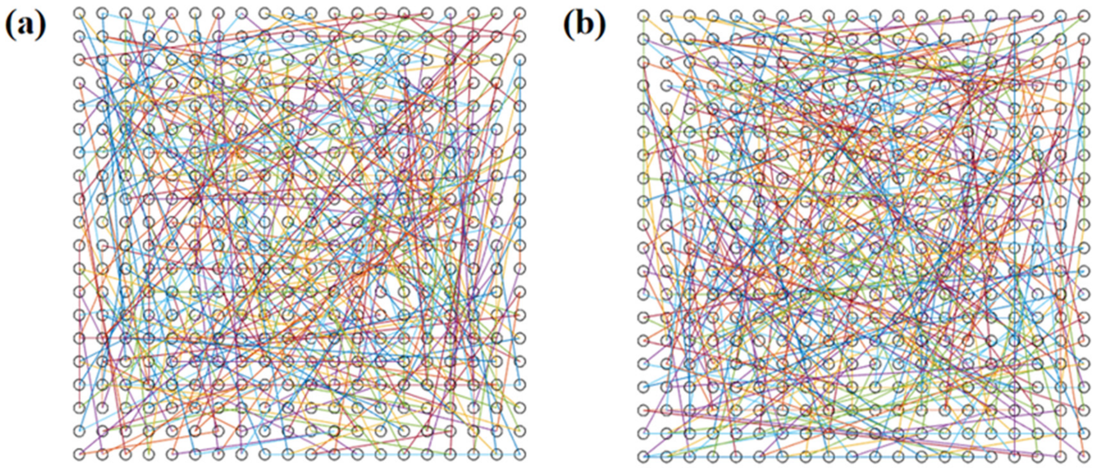

Figure S1. Schematic diagrams of SAA paths under different constraints R=6. (a) $M = 1$; (b) $M = 2$.

In Figure S2, it illustrates how the minimum flying distance L changes with spacing-point number M for fixed $R = 6$. The minimum flying distance L increases as M becomes larger.

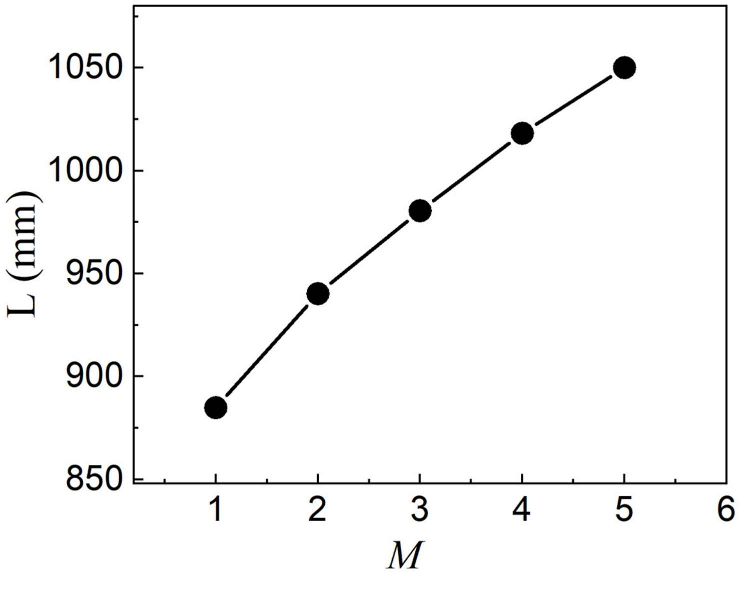

Figure S2. Trend of minimum flying distance L against spacing-point number M ($R = 6$).

S2. Experimental results and Analysis

S2.1. The effect of laser power P on the hole quality parameters

In this section, when the $v = 100$ mm/s and $N = 1$, the quality parameters (h, w, d, D) about the PMMA hole array as a function of laser power P under the SSM and SAA paths are shown in Figure S3. The four parameters h, w, d, D all increase as the laser power P increases both under the SSM and SAA paths. The greater the laser power is, the more energy is transferred to the material, leading to an increase in these hole parameters. As illustrated in Figure S3(a), (c) and (d), it is evident that both under the SSM and SAA paths, the height h about the molten deposits, the depth d and diameter

D of the hole are similar. However, in Figure S3(b), the width *w* of the molten deposits behaves differently between the SSM and SAA paths. When $P \leq 3$ W, there is negligible variation in the width *w*. But when $P > 3$ W, the width *w* under the SAA path is significantly smaller than that under the SSM path. Table I displays the widths *w* of molten deposits when laser power $P = 3.6$ W and 4.5 W. Notably, at $P = 4.5$ W, the width *w* under the SAA path can be reduced by about 18% compared to the SSM path.

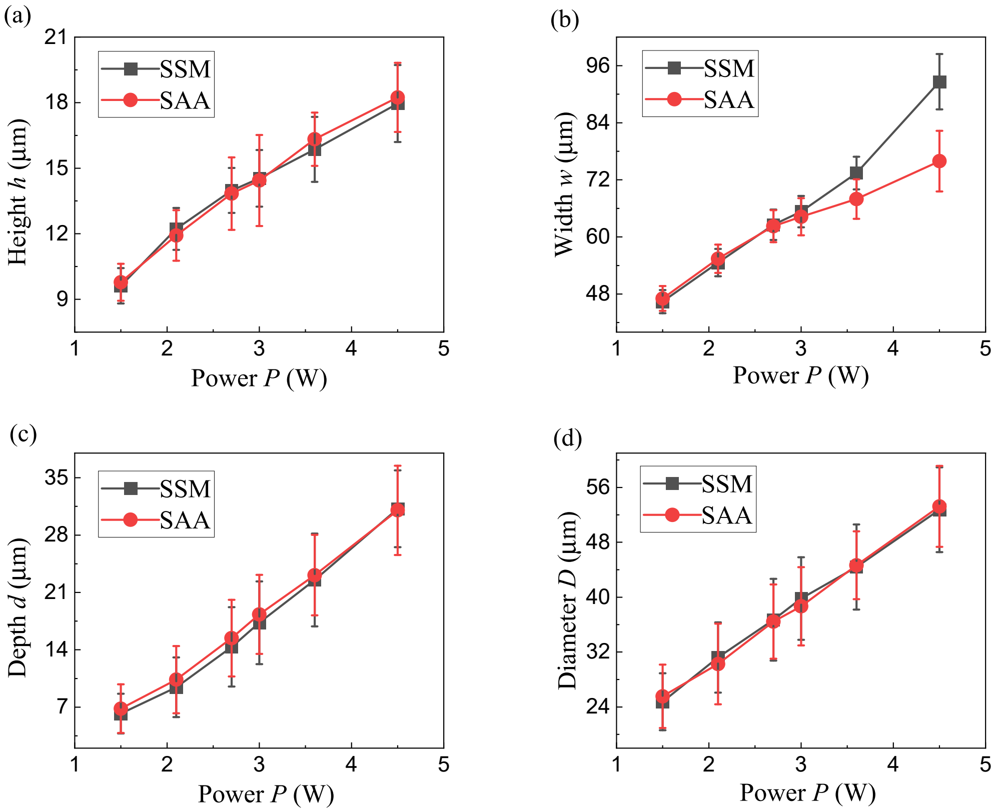

Figure S3. At the conditions of the $v = 100$ mm/s and $N = 1$, the effect of laser power *P* on the hole quality parameters under the SSM and SAA paths: (a) The height *h* and (b) width *w* of the molten deposits around single hole; (c) The depth *d* and (d) diameter *D* of the single hole.

S2.2. The effect of scanning speed v on the hole quality parameters

In this section, when the $P = 3$ W and $N = 1$, the quality parameters (*h*, *w*, *d*, *D*) about the PMMA hole array as a function of scanning speed *v* under the SSM and SAA paths are shown in Figure S4.

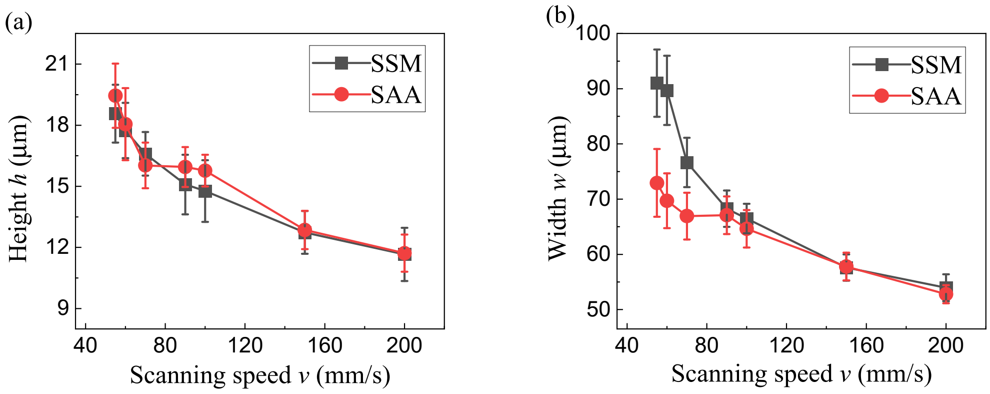

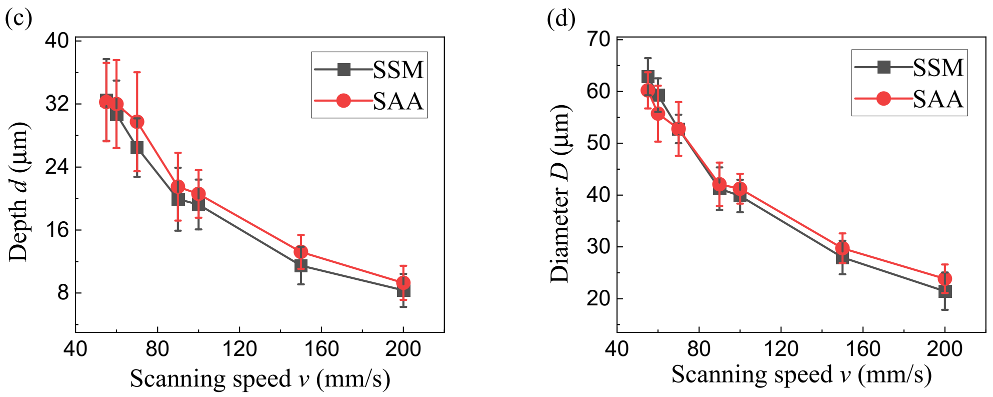

Figure S4 At the conditions of the $P = 3$ W and $N = 1$, the effect of scanning speed v on the hole quality parameters under the SSM and SAA paths: (a) The height h and (b) width w of the molten deposits around single hole; (c) The depth d and (d) diameter D of the single hole.

As illustrated in Figure S4, there is a decrease in the four parameters h, w, d, and D as the scanning speed v increases under the SSM and SAA paths. This decrease can be attributed to the reduced overlap rate of laser pulse at higher scanning speed[38], which in turn lowers the laser energy density acting on the materials. It can be seen from Figure S4(a) and (d), there is an insignificant difference in the height h of the molten deposits and the diameter D of the hole both under the SSM and SAA paths. In Figure S4(c), it can be found that the single hole under the SAA path is slightly deeper than that under the SSM path. However, Figure S4(b) demonstrates a distinct difference in the width w of the molten deposits under the two paths. When $v > 70$ mm/s, the widths w remain comparable, but when $v \leq 70$ mm/s, the width w under the SAA path is obviously narrower than that under the SSM path.

S2.3. The effect of scanning number N on the hole quality parameters

In this section, when the $P = 3$ W and $v = 150$ mm/s, the quality parameters (h, w, d, D) about the PMMA hole array as a function of scanning number N under the SSM and SAA paths are shown in Figure S5.

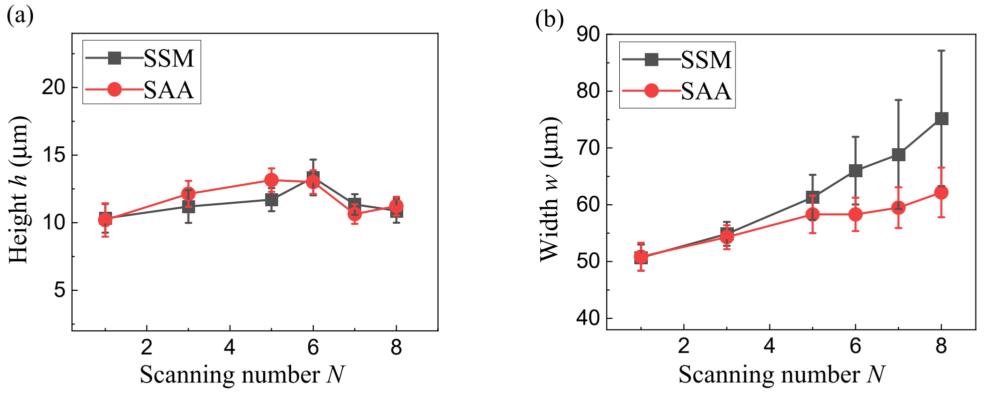

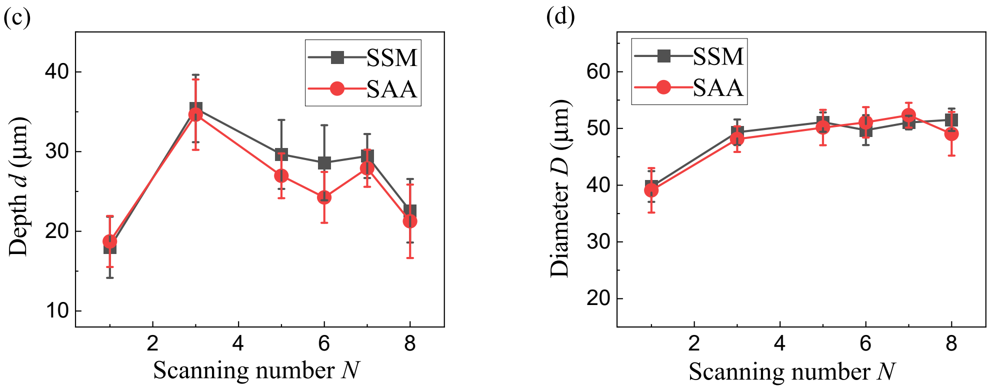

Figure S5 At the conditions of the P = 3 W and v = 100mm/s, the effect of scanning number N on the hole quality parameters under the SSM and SAA paths: (a) The height h of and (b) width w of the molten deposits around single hole; (c) The depth d and (d) diameter D of the single hole.

As illustrated in Figure S5, there is insignificant variation observed in the height h of the molten deposits, as well as in the depth d and diameter D of the hole, irrespective of whether the processing is conducted under the SSM path or the SAA path. Specifically, in Figure S5(a), it is evident that h stabilizes around 12 μm. In Figure S5(b), as the scanning number N increases, the widths w of the molten deposits gradually increase both under the SSM and SAA paths. Hole depth and diameter show little dependence on scanning strategy, with only negligible differences observed.
